# Characterization and phylogenetic analysis of the chloroplast genome of *Iris lactea* var. *chinensis*

**DOI:** 10.1080/23802359.2020.1847611

**Published:** 2021-04-27

**Authors:** Xiaoran Cai, Bin Zhang, Shuai Wang, Yueqin Cheng, Hongwei Wang

**Affiliations:** a College of Plant Protection, Henan Agricultural University, Zhengzhou, China; b College of Life Science, Henan Agricultural University, Zhengzhou, China

**Keywords:** *Iris lactea* var. *chinensis*, chloroplast genome, phylogenetic analysis

## Abstract

*Iris lactea* var. *chinensis* is a well-regarded ornamental plant in the genus *Iris* (family Iridaceae). In this report, we present the complete chloroplast (cp) genome sequence of *I. lactea* var. *chinensis* for the first time. The complete cp genome of *I. lactea* var. *chinensis* was assembled using high-throughput sequencing, and phylogenetic analysis was undertaken based on a dataset of coding regions. The cp genome of *I. lactea* var. *chinensis* measures 152,409 bp in length, with regions having two inverted copies (IR 26,026 bp), and separated by the large single copy (LSC 82,256 bp) and small single copy (SSC 18,101 bp) regions. The cp genome encodes 133 unique genes, including 87 different protein-coding genes, 38 tRNA genes, and 8 rRNA genes. Based on a dataset of 69 chloroplast coding regions, the maximum-likelihood (ML) phylogenetic tree analysis indicated that *Iris lactea* var. *chinensis* clusters closely with *Iris sanguinea*. Thus, the complete chloroplast genome presented in this report may provide valuable genetic information not only for the future exploitation and utilization of this plant resource but also for further research investigating its relationship with other *Iris* species.

*Iris lactea* var. *chinensis* is a perennial herb of the genus *Iris* (family Iridaceae) native to China that is widely distributed in Northeast China, North China, and Northwest China. This species is one of the most valuable iris plants (Xu et al. 2011). Because the flowers of this species exhibit good appearance and gorgeous colors, it has high ornamental value and can be planted on the edges of garden roads, flower beds and flower borders, embellished in lawns, or directly used as ground cover plants (Tang et al. 2018). *I. lactea* var. *chinensis* has a well-developed root system and strong drought resistance; therefore, it is also a useful sand-fixation and greening plant. In addition, as a halophyte, *I. lactea* var. *chinensis* has strong salt resistance and can be used in the improvement of saline land (Gu et al. 2018). *I. lactea* var. *chinensis* also showed strong Cd tolerance and accumulation ability, indicating significant potential for application in the phytoremediation of Cd-contaminated soil (Gu et al. 2017; Tian et al. 2019; Liu et al. 2020). In this study, for the first time, we report the chloroplast (cp) genome of this species based on Illumina HiSeq paired-end sequencing data, which may provide valuable genetic information not only for the future exploitation and utilization of this plant resource but also for further research investigating its relationship with other *Iris* species.

The leaf sample of *Iris lactea* var. *chinensis* was collected from Zhengzhou (34°48′3″N, 113°48′44″E), Henan Province, China. The voucher specimen (IRII20190026) is kept in the herbarium of Henan Agricultural University. Total genomic DNA was extracted using a modified CTAB method (Stefanova et al. 2013). The high-quality DNA was cleaved, and paired-end library preparation and sequencing were performed on an Illumina HiSeq platform. The raw data were quality filtered at a Phred score < 30. All remaining sequences were assembled into contigs using NOVOPlasy-v3.3 (Dierckxsens et al. 2017) to reconstruct the cp genome, with *Iris sanguinea* (GenBank accession number: NC_029227) serving as a reference. The annotation and correction of the cp genome were performed through the program Geneious Prime (Kearse et al. 2012). This genome sequence was deposited into GenBank (accession number MT740331).

The complete cp genome of *I. lactea* var. *chinensis* measures 1,52,409 bp in length, containing a large single-copy (LSC) region of 82,256 bp, a small single-copy (SSC) region of 18,101 bp, and two inverted repeat (IR) regions of 26,026 bp. The overall GC content of the whole plastid genome is 38.0%, whereas the corresponding values of the LSC, SSC, and IR regions are 36.2%, 31.7%, and 42.9%, respectively. The whole sequence of *I. lactea* var. *chinensis* contains 133 genes, including 87 protein-coding genes, 38 tRNA genes, and 8 rRNA genes. Among these genes, 20 are duplicated, including eight protein-coding genes (*ndhB*, *rpl2*, *rpl23*, *rps12*, *rps7*, *rps12*, *rps19* and *ycf2*), four ribosomal RNA genes, and eight transfer RNA genes (*trnA-UGC*, *trnH-GUG*, *trnI-CAU*, *trnI-GAU*, *trnL-CAA*, *trnN-GUU*, *trnR-ACG*, *trnV-GAC*). In addition, twenty-three of the genes contain one or two introns.

To perform phylogenetic analysis, a dataset of 69 chloroplast coding regions of 11 species, including six species from Iridaceae and five species from other families in Asparagales, was aligned using the MAFFT method in Geneious Prime. The maximum likelihood (ML) phylogenetic tree was constructed using RAxML-HPC2 on XSEDE v8.2.12 on the CIPRES cluster (Miller et al. 2010). The ML phylogenetic results indicated that six species from Iridaceae clustered into one branch, which was sister to another branch containing three species from Amaryllidaceae, Asparagaceae and Asphodelaceae (Figure 1). Four *Iris* species formed a monophyletic group, in which *I. gatesii* was the earliest species to diverge, and *I. lactea* var. *chinensis* was determined to be closely related to *I. sanguinea*.

**Figure 1.**
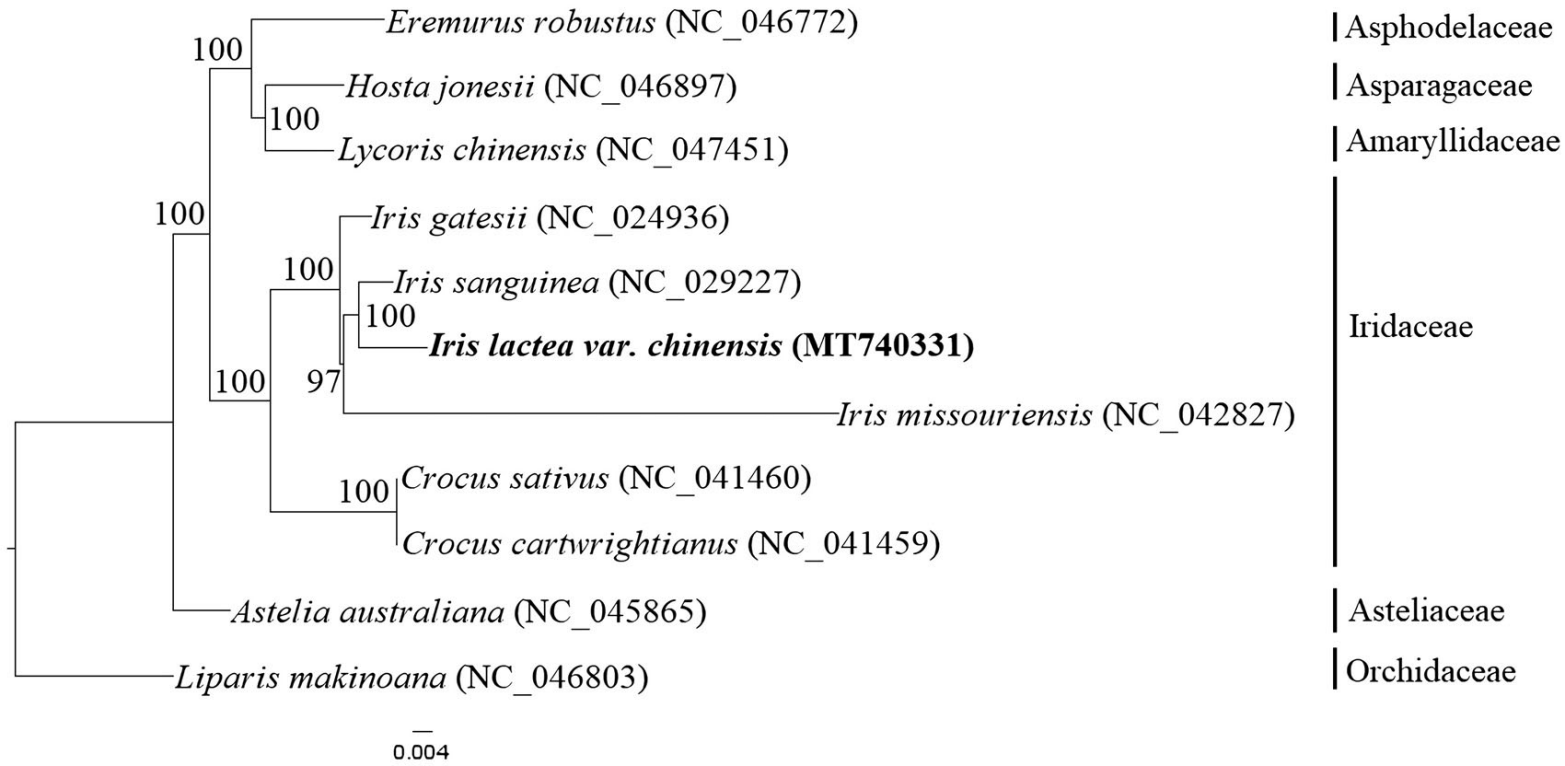
ML phylogenetic tree reconstruction including 11 species based on a dataset of 69 chloroplast coding regions. Bootstrap support values are shown beside the nodes. The new complete chloroplast genome obtained in this study is shown in bold.

## Data Availability

The data that support the findings of this study are publicly available in the National Center for Biotechnology Information (NCBI) at https://www.ncbi.nlm.nih.gov, accession number MT740331.
